# Increased adipose tissue lymphatic vessel density inhibits thermogenesis through elevated neurotensin levels

**DOI:** 10.3389/fcell.2023.1100788

**Published:** 2023-01-27

**Authors:** Thien T. Phan, Adri Chakraborty, Madison A. Tatum, Ana Lima-Orellana, Andrea J. Reyna, Joseph M. Rutkowski

**Affiliations:** ^1^ Department of Medical Physiology, Texas A&M University School of Medicine, Bryan, TX, United States; ^2^ Currently the Arthritis and Autoimmune Disease Research Center, Boston University School of Medicine, Boston, MA, United States

**Keywords:** lymphangiogenesis, thermogenesis, UCP-1, high fat diet, neurotensin. (Min.5-Max. 8)

## Abstract

During cold exposure, white adipose tissue can remodel to dissipate energy as heat under cold similar to thermogenic brown adipose tissue. This “browning” and the regulation of body temperature is under the control of neural and hormonal signaling. It was recently discovered that neurotensin, a small neuropeptide, not only acts to inhibit thermogenesis, but also that lymphatic vessels may be a surprisingly potent source of neurotensin production. We hypothesized that the induction of adipose tissue lymphangiogenesis would therefore increase tissue neurotensin levels and impair thermogenesis.

**Methods:** We utilized AdipoVD mice that have inducible expression of vascular endothelial growth factor (VEGF)-D, a potent lymphangiogenic stimulator, specifically in adipose tissue. Overexpression of VEGF-D induced significant lymphangiogenesis in both white and brown adipose tissues of AdipoVD mice.

**Results:** Obese Adipo-VD mice demonstrated no differences in adipose morphology or browning under room temperature conditions compared to controls but did express significantly higher levels of neurotensin in their adipose tissues. Upon acute cold exposure, AdipoVD mice were markedly cold intolerant; inhibition of neurotensin signaling ameliorated this cold intolerance as AdipoVD mice were then able to maintain body temperature on cold challenge equivalent to their littermates.

**Conclusion:** In total, these data demonstrate that adipose tissue lymphatic vessels are a potent paracrine source of neurotensin and that lymphangiogenesis therefore impairs the tissues’ thermogenic ability.

## Introduction

Adipose tissue remodeling is essential for the tissue to maintain its normal physiological roles in caloric storage and temperature maintenance (Kusminski et al., 2016; Scherer, 2019). White adipose tissue (WAT) expands with excess calories and brown adipocytes generate warmth through thermogenesis (Rosen and Spiegelman, 2014; Shinde et al., 2021). Healthy white adipose tissue also demonstrates the ability to “beige” during cold exposure and thus generate heat through adaptive thermogenesis. In the pandemic of obesity, aberrant WAT remodeling with weight gain results in adipose tissue inflammation, immune cell infiltration, and cytokine and lipid spillover to the patient’s body leading to the metabolic syndrome (Medzhitov, 2008; Rutkowski et al., 2009; Sun et al., 2011; Sun et al., 2013; Rutkowski et al., 2015; Crewe et al., 2017). WAT adaptive thermogenesis is also impaired with obesity and inflammation (Petrovic et al., 2010). Numerous preclinical models have attempted to correct this progression by augmenting adipose blood vasculature, altering fibrosis, or manipulating the inflammatory response to improve WAT remodeling, tissue health, and ameliorating the metabolic dysfunction seen with obesity (Rutkowski et al., 2009; Sun et al., 2011; Sun et al., 2013).

The lymphatic system helps to maintain homeostasis through interstitial fluid drainage, transport of macromolecules (e.g., lipids, antigens, and cytokines), immune cell transport, and direct and indirect immunomodulation (Wiig and Swartz, 2012; Card et al., 2014; Maisel et al., 2017). This makes lymphatic vessels and lymphatic endothelial cells (LECs), central to the resolution of tissue inflammation (Abouelkheir et al., 2017). Lymphangiogenesis is characteristic of acute and chronic inflammation as a biological response to elevated growth factor levels—notably vascular endothelial growth factors -C and -D (VEGF-C; VEGF-D)–that can aid in decreasing fluid and immune accumulation and increasing immune transport from the tissue (Abouelkheir et al., 2017). We have previously demonstrated that inducible overexpression of VEGF-D in adipose tissue of mice results in a dense adipose lymphatic network, improved glucose metabolism, and increased immune trafficking from adipose tissue (Chakraborty et al., 2019; Chakraborty et al., 2020). These “AdipoVD mice” demonstrated that adipose lymphatic transport and immune roles may be a target in regulating adipose tissue to improve metabolic outcomes.

Aside from the classical roles of fluid drainage and immune regulation of lymphatic vessels, several studies have demonstrated that LECs impact the tissue microenvironment through newly-discovered paracrine roles (Liu et al., 2020; Li et al., 2021; Niec et al., 2022; Yoon and Detmar, 2022). Li and others recently described LECs as a critical source of neurotensin, a neuropeptide for which the authors characterized a novel function as an anti-thermogenic factor (Li et al., 2021). Using LEC-specific deletion of neurotensin in mice, the authors demonstrated that adipose tissue thermogenesis was improved along with overall energy consumption. The authors also posited that lymphatics are largely absent from brown adipose tissue due to the potential anti-thermogenic effect of LECs (Li et al., 2021).

Based on this novel finding of a potential LEC paracrine role, we hypothesized that adipose lymphangiogenesis would provide an additional source of neurotensin and thus adversely impact adaptive thermogenesis. To test whether lymphatic vessels impede thermogenesis through neurotensin we utilized the AdipoVD mouse to compare mice with normal and augmented adipose lymphatic density in their cold response. Importantly, these mice have previously demonstrated a strong lymphangiogenic response in both the brown and subcutaneous WAT necessary for thermogenesis (Chakraborty et al., 2019). We identify increased neurotensin in AdipoVD mouse lymphatic-dense adipose tissues and demonstrate a diminished thermogenic response that could be corrected with neurotensin inhibition.

## Materials and methods

### Animals

AdipoVD mice with expansion of LEC density specifically in adipose tissue were utilized as previously described (Chakraborty et al., 2019; Chakraborty et al., 2020). A new cohort of AdipoVD mice that were hemizygous for both TRE-VEGF-D and *AdipoQ-*rtTA transgenes were utilized while their littermates with only one transgene were utilized as controls for all experiments. All mice were age-matched and mixed sexes were utilized. All mice were housed in an AALAC-approved facility with a 12-h light-dark cycle with *ad libitum* access to food and water. All mice received diets with doxycycline (600 mg/kg food), to account for any doxycycline side effects, in a high fat diet consisting of 60% kcal of fat [D16042102, lard based (supplemented D12492)] for 16 weeks. The mice were approximately 8–12 weeks of age at the start of the diet. All euthanasia was performed by cervical dislocation following exsanguination under deep isoflurane (>5%). The Institutional Animal Care and Use Committee at Texas A&M University (College Station, TX) approved all animal study procedures.

#### Body composition

Mouse body composition was measured 1 week before euthanasia to measure the percentage of lean, fat, and fluid mass relative to overall mass by EchoMRI 100H (EchoMRI LLC, Houston, TX).

### Neurotensin receptor inhibition

The neurotensin receptor 2 inhibitor NTRC 824 (Tocris Bioscience, Minneapolis, MN) was delivered by intraperitoneal injection for 5 days at 5 mg/kg body weight daily in 10% dimethyl sulfoxide and phosphate buffered saline solution. Cold exposure experiments were performed immediately following the fifth and final injection.

#### Cold exposure

All mice were placed in individual housing and initial body temperatures were measured by a rectal thermometer probe prior to cold exposure. Cages were then placed into a 4°C cold room with body temperatures measured every 30 min. *Ad libitum* access to food and water was provided. Mice were immediately removed from the cold room if their body temperature dropped below 26°C.

### Tissue collection

Immediately following cold exposure (after 4 h of cold exposure or when body temperature reached 26°C), mice were exsanguinated under deep isoflurane and adipose tissues were harvested and either fixed in 10% buffered zinc formalin for histology or flash frozen for protein and RNA isolation. The inguinal lymph node was removed from the subcutaneous inguinal WAT (SQAT) prior to excision such that only adipose tissue proper was assessed further and the interscapular brown adipose tissue (BAT) was cleaned of its surrounding white adipose tissue for clear analyses of BAT-only expression.

### RNA isolation and quantitative PCR

To remove the abundant lipids from adipose tissues the tissues were first homogenized in Trizol and then centrifuged. After removal of the lipid layer, the tissue pellets were resuspended and mixed with chloroform before an additional centrifugation. The Zymo Direct-zol RNA Miniprep Plus kit was used to isolate RNA (Zymo Research, Irvine, CA). Reverse transcription of 1000 ng RNA was done with the Bio-Rad iScript cDNA Synthesis Kit (Bio-Rad Laboratories, Inc., Hercules, CA). Quantitative real time PCR was performed on ​​a 384-well QuantStudio 6 Flex quantitative PCR machine (Applied Biosystem, Foster City, CA) using iTaq universal SYBR Green Master Mix (Bio-Rad Laboratories, Inc.). The primer sequences utilized for amplification are listed in Table 1. Data are presented as normalized to the control mouse tissues for each respective tissue.

**TABLE 1:** Primers and sequences utilized in this study.

Genes	Forward	Reverse
*Cox7a*	GCT CTG GTC CGG TCT TTT AGC	GTA CTG GGA GGT CAT TGT CGG
*Cox7a1*	GCT CTG GTC CGG TCT TTT AGC	GTA CTG GGA GGT CAT TGT CGG
*Pdk4*	AGG GAG GTC GAG CTG TTC TC	GGA GTG TTC ACT AAG CGG TCA
*Neurotensin (Nts)*	GTG TGG ACC TGC TTG TCA GA	TCA TGC ATG TCT CCT GCT TC
*Neurotensin Receptor 1 (Ntsr1)*	TCT GAT GTT GGA CTT GGG TTC	AGT GCT ATG GTA TCT GCT GG
*Neurotensin Receptor 2 (Ntsr2)*	TCT CTC AGT TCC CTG TGT GG	AGC CAT TGT TTG TTC TC
*Neurotensin receptor 3 (Ntsr3/Sort1)*	TTC CCA GAC TAT CCT CAC CC	TAT TGA CCA CAC ACG GCA TC
*Prdm16*	ACA CGC CAG TTC TCC AAC CTG T	TGC TTG AGG GAG GTA
*Cox8b*	TGC TGG AAC CAT GAA GCC AAC	AGC CAG CCA AAA CTC CCA CTT
*Dio2*	CAT TGA GGC TCA CCC TTC	GGT TCC GGT GCT TCT TAA CCT
*Ppargc1a*	GCA​CCA​GAA​AAC​AGC​TCC​AAG	CGT​CAA​ACA​CAG​CTT​GAC​AGG
*Prox1*	AGA​AGG​GTT​GAC​ATT​GGA​GTG​A	TGC​GTG​TTG​CAC​CAC​AGA​ATA
*Ubc*	GCC​CAG​TGT​TAC​CAC​CAA​GAA​G	GCT​CTT​TTT​AGA​TAC​TGT​GGT​GAG​GAA
*Ucp1*	TCT​CAG​CCG​GCT​TAA​TGA​CTG	GGC​TTG​CAT​TCT​GAC​CTT​CAC

#### Histology and immunofluorescence

Tissues were fixed for 24 h and paraffin embedded prior to sectioning for immunofluorescence labeling. Tissue sections were deparaffinized by xylene, rehydrated with serial ethanol steps, and permeabilized with 0.1% Triton solution in PBS. Tissues were blocked with 20% Aqua Block (East Coast Bio) solution in PBS and incubated with primary antibodies against Neurotensin (14670S, Cell Signaling Technology), UCP-1 (ab233107, Abcam), Lyve1 (AF2125, R&D Systems), or Endomucin (sc-65495, Santa Cruz) overnight. Following fluorescent secondary antibody labelling, tissue sections were mounted with DAPI Fluoromount-G (SouthernBiotech, Birmingham, AL). Representative images were taken with an Olympus BX51 fluorescence microscope and Olympus Q5 camera using CellSens Standard Version 1.9 software.

#### Protein isolation and quantitation

Tissues were homogenized in 50 mmol/L of Tris, 150 mmol/L NaCl, and 1 mmol/L EDTA containing HALT protease and phosphate inhibitor (ThermoFisher, Waltham, MA). After centrifuging the homogenate and removing the fat cake, additional buffer with 10% Triton X-100 was added to pellet (for a final of 1% Triton X-100) and tissue homogenate was re-homogenized. Pierce BCA Protein Assay (ThermoFisher, Waltham, MA) was utilized to quantify total protein concentrations. Neurotensin protein levels from tissue lysates were measured by Neurotensin EIA kit (RayBiotech) according to manufacturer’s protocols and normalized to the total protein concentration.

### Statistical analysis and data presentation

Sample size utilized in this study were as follows: n = 11 control and AdipoVD for examining relative gene expression without temperature challenge and n = 10 control/n = 7 AdipoVD for temperature challenge experiment. Statistical analyses were performed utilizing paired two tailed *t*-test in comparing changes in body temperature and unpaired two tailed *t*-test for comparison of gene expression and ELISA quantification. All data are presented as means ± SD except for the time course temperature plots that utilize SE only for the sake of visual clarity on the graph. *p* < 0.05 was considered significant.

## Results

### Adipose lymphangiogenesis does not significantly alter adiposity or adipose browning during high fat feeding

**FIGURE 1:**
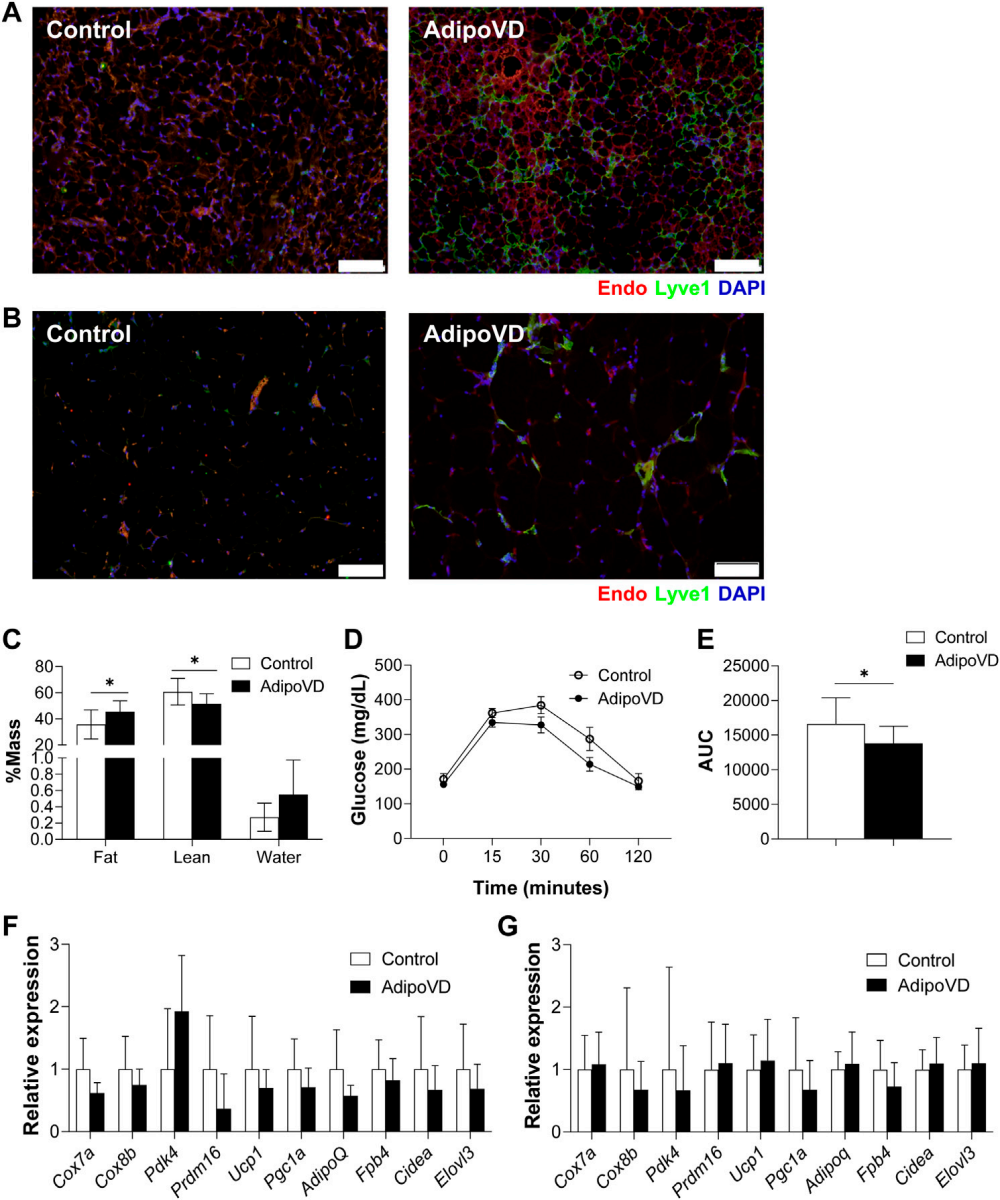
Overexpression of VEGF-D expands adipose lymphatic density but does not impact overall adipose tissue physiology. **(A)**: Immunofluorescence of lymphatic (green; LYVE-1) and blood (red; Endomucin) in brown adipose tissue (BAT) of Control and AdipoVD mice. **(B)** Immunofluorescence of lymphatic (green; LYVE-1) and blood (red; Endomucin) in subcutaneous white adipose tissue (SQAT) of Control and AdipoVD mice. **(C)** Body composition quantification of Control and AdipoVD mice by magnetic resonance. **(D)** Serum glucose levels during an oral glucose tolerance test in Control and AdipoVD mice. **(E)** Average area under the curve (AUC) calculated from glucose levels of oral glucose tolerance test. **(F)** Browning and thermogenesis-associated gene expression in AdipoVD BAT relative to Controls. **(G)** Browning and thermogenesis-associated gene expression in AdipoVD SQAT relative to Controls. **(A–E)** *n* = 10 control/n = 7 AdipoVD. **(F–G)**; *n* = 11 for control and AdipoVD. Blue = DAPI. Bars = 100 µm.

Lymphatic vessels in adipose tissue of mice and man are relatively sparse compared to other vascularized tissues (Chakraborty et al., 2020; Redondo et al., 2020; Varaliova et al., 2020). To manipulate adipose tissue lymphatic density in mice, we overexpressed VEGF-D in adipose tissue in the previously characterized AdipoVD mouse model for 4 months (Chakraborty et al., 2020). Adipose tissue sections demonstrated that AdipoVD mice have a markedly increased lymphatic density in both the subcutaneous WAT (SQAT; Figure 1A) and interscapular brown adipose tissue (BAT; Figure 1B) in comparison, to controls that which largely lack any lymphatics. Gonadal white adipose tissue (GWAT) demonstrated minimal lymphatic expansion (Supplemental Figure 1A). Similar to our previous study, obese AdipoVD mice demonstrated a small, but significant increase in fat mass (and less lean mass) compared to controls (Figure 1C) (Chakraborty et al., 2019). Obese AdipoVD mice also demonstrated enhanced glucose clearance when tested by an oral glucose tolerant test (Figure 1D; E). At normal facility housing temperature, there were no significant differences in the RNA expression of common brown and ‘browning’ genes in the BAT of AdipoVD mice compared to controls (Figure 1F). No significant differences were found in the SQAT of AdipoVD mice for the same gene panel compared to controls (Figure 1G). VEGF-D overexpression and adipose tissue lymphangiogenesis therefore do not profoundly affect the overall adiposity or browning of adipose tissue in obese AdipoVD mice when studied at room temperature.

### Mice with increased adipose lymphatic density demonstrate elevated neurotensin levels

**FIGURE 2:**
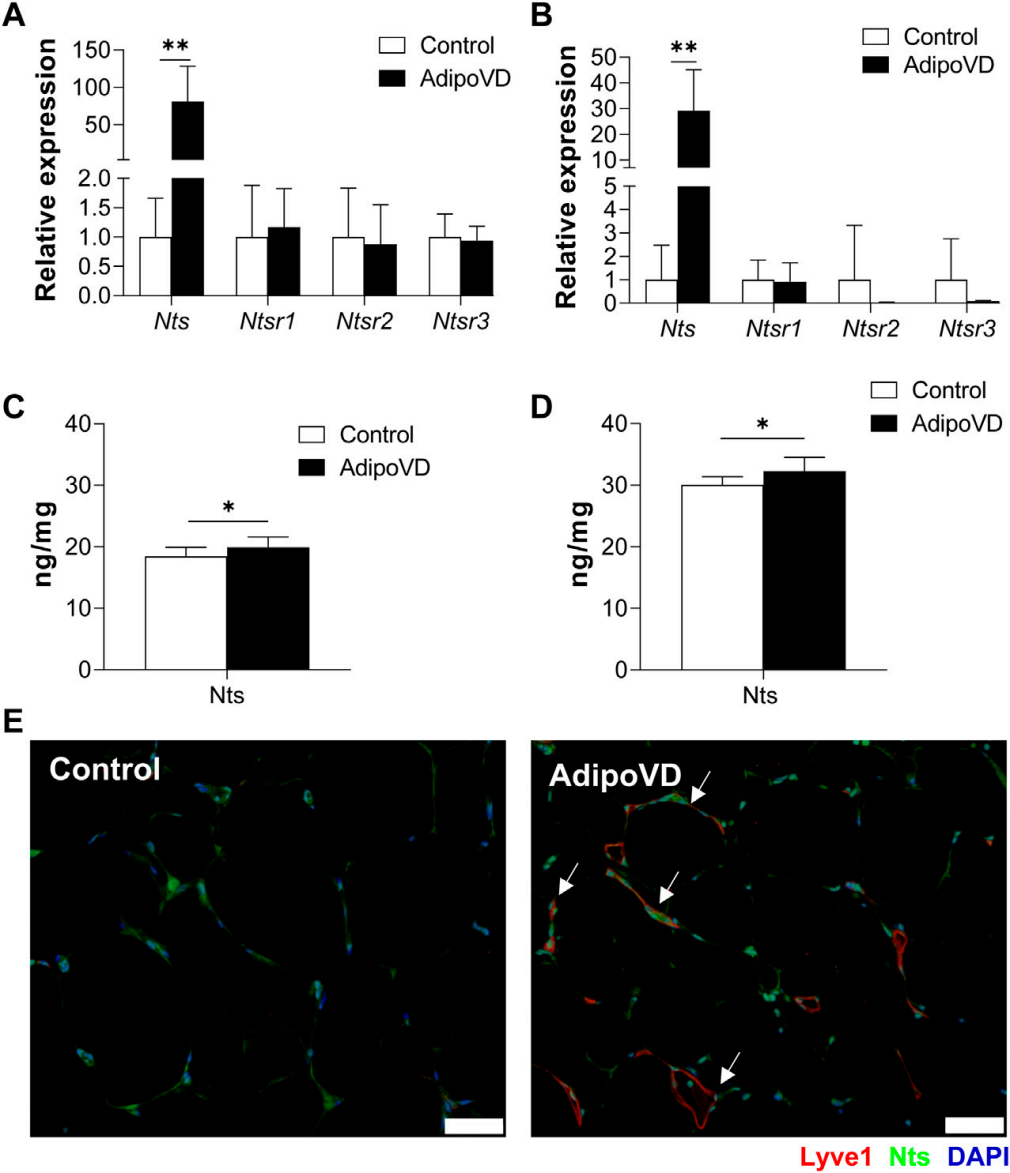
Lymphangiogenesis in AdipoVD mice result in increased neurotensin production. **(A)** Neurotensin and neurotensin receptor relative mRNA expression in BAT of AdipoVD mice compared to Controls. **(B)** Neurotensin and neurotensin receptor relative mRNA expression in SQAT of AdipoVD mice compared to Controls. **(C)** ELISA quantification of neurotensin concentrations from BAT normalized to tissue protein levels. **(D)** ELISA quantification of neurotensin concentrations from SQAT normalized to tissue protein levels. **(E)** Immunofluorescence of neurotensin (green; Nts) and lymphatics (red; LYVE-1) in the BAT depot from Control and AdipoVD mice on HFD. **(A–E)** *n* = 10 control/*n* = 7 AdipoVD. Bars = 50 µm.

If LECs are an important source of neurotensin secretion, AdipoVD mice with a dense adipose lymphatic network should have elevated neurotensin levels. Indeed, AdipoVD mice exhibited significantly increased *Neurotensin* (*Nts*) mRNA expression in both BAT (Figure 2A) and SQAT (Figure 2B). In GWAT, where lymphatic expansion was minimal, the elevation of *Nts* expression was not significant (Supplementary Figure 1B). The gene expression of neurotensin receptors 1, 2, and 3 were not significantly different in any of the AdipoVD adipose depots compared to control mice (Figures 2A, B; Supplementary Figure 1B).

To confirm that gene expression resulted in increased protein levels, neurotensin tissue concentrations were measured by ELISA. Both BAT and SQAT depots had significantly increased neurotensin protein concentrations in AdipoVD mice compared to their littermates (Figures 2C, D). Interestingly, overall neurotensin concentrations were higher in the SQAT depot in comparison to BAT depot seen in both Control and AdipoVD mice; with Nts concentrations in SQAT at 30.3 ± 1.46 ng/mg in Control vs 32.5 ± 2.27 ng/mg in AdipoVD compared to BAT concentrations at 18.5 ± 1.44 ng/mg in Control vs 19.9 ± 1.66 ng/mg in AdipoVD. Intriguingly, the new LEC contribution to overall neurotensin quantity is relatively small at a few ng/mg in AdipoVD mice. Immunofluorescence labeling of neurotensin identified co-localization of LYVE-1 and Nts in SQAT depot, however, labeling also indicated that other non-lymphatic cells also produce neurotensin (Figure 2E). Altogether, these data establish that neurotensin expression and production increased as a result of lymphangiogenesis within adipose tissue.

### Mice with increased adipose lymphatic density demonstrate cold intolerance

**FIGURE 3:**
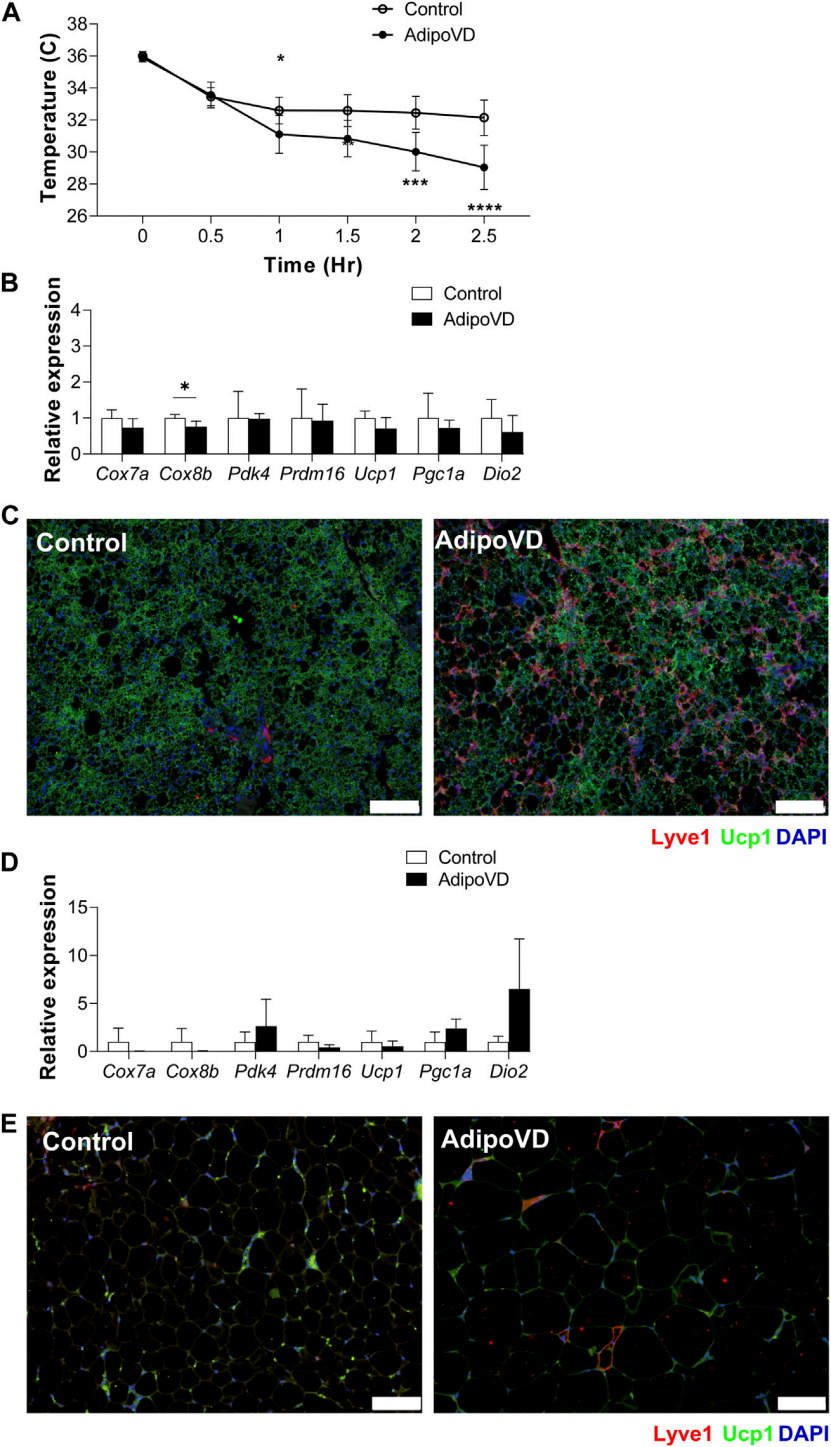
Temperature dysfunction in AdipoVD mice as a result of increased neurotensin levels. **(A)** Body temperature of Control and AdipoVD mice during acute under cold exposure at 4°C. **(B)** Browning and thermogenesis-associated gene expression in AdipoVD BAT relative to Controls under acute cold exposure. **(C)** Immunofluorescence of potential thermogenesis (green; UCP-1) and lymphatic (red; LYVE-1) from BAT depot of Control and AdipoVD following cold exposure. **(C)** Immunofluorescence of UCP-1 (green) and lymphatics (red; LYVE-1) from BAT depot of Control and AdipoVD following cold exposure. **(D)** Browning and thermogenesis-associated gene expression in AdipoVD SQAT relative to Controls following cold exposure. **(E)** Immunofluorescence of potential thermogenesis (green; UCP-1) and lymphatic (red; LYVE-1) from SQAT depot of Control and AdipoVD following cold exposure. **(A–E)** *n* = 10 control/*n* = 7 AdipoVD. Blue = DAPI. Bars = 100 µm.

Neurotensin was well-characterized as an anti-thermogenic neuropeptide by Li and others (Li et al., 2021). To test if the elevated neurotensin levels in lymphatic-dense adipose tissue impacted the thermogenic response in AdipoVD mice, we performed acute cold exposure experiments at 4°C. AdipoVD mice core body temperature decreased after 1 h of cold exposure and continued to rapidly decrease up to 2.5 h of cold exposure in comparison to the more gradual cooling in control mice (Figure 3A). This rapid temperature decline necessitated removal of AdipoVD mice earlier than planned from the cold. Post-cold exposure, the BAT depot had no significant differences in thermogenesis-associated genes measured except for *Cox8b* being significantly lower when compared to cold controls (Figure 3B). Immunofluorescence labeling of uncoupling protein 1 (UCP-1) demonstrated labeling in the BAT of both control and AdipoVD mice with no obvious regionality based on lymphatic density (Figure 3C). In the SQAT depot, *Dio2*, *Pdk4,* and *Pgc1a* had higher relative expression levels, however, none were significant (Figure 3D). “Browning”, based on detection of UCP-1 expression in the SQAT depot by immunofluorescence, was not remarkable in either control of AdipoVD mice, likely due to the short timeframe of the experiment (Figure 3E). The elevated neurotensin levels resulting from adipose tissue lymphangiogenesis thus negatively impacted thermogenesis during cold exposure in our AdipoVD mice.

### Neurotensin inhibition eliminates the anti-thermogenic effects of increased lymphatic density

**FIGURE 4:**
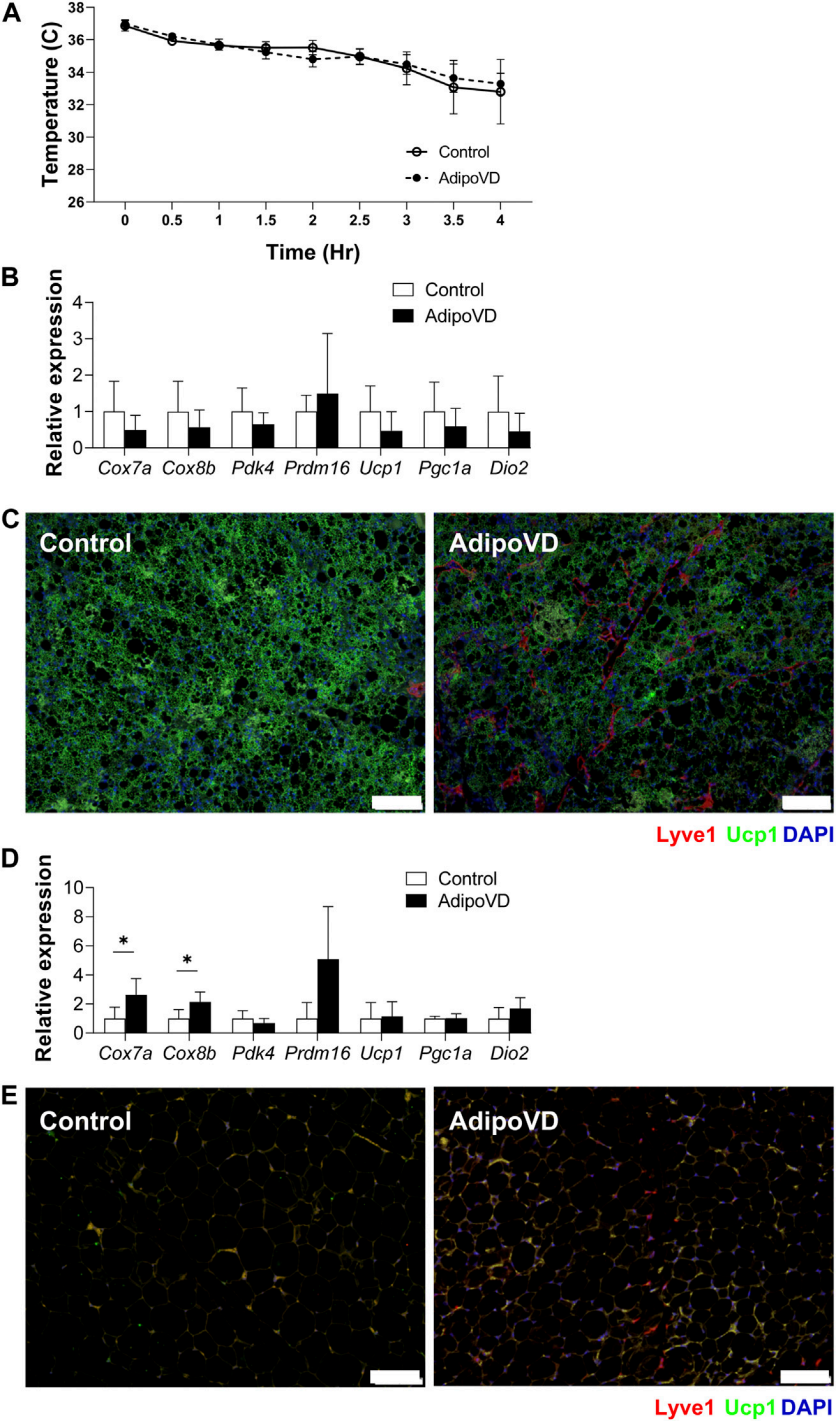
Inhibition of neurotensin activity ameliorate temperature dysfunction in AdipoVD mice. **(A)** Body temperature of Control and AdipoVD mice during acute under cold exposure at 4°C after neurotensin receptor inhibition. **(B)** Browning and thermogenesis-associated gene expression in AdipoVD BAT relative to Controls under acute cold exposure after neurotensin receptor inhibition. **(C)** Immunofluorescence of UCP-1 (green) and lymphatics (red; LYVE-1) from BAT depot of Control and AdipoVD following cold exposure. **(D)** Browning and thermogenesis-associated gene expression in AdipoVD SQAT relative to Controls following cold exposure and neurotensin receptor inhibition. **(E)** Immunofluorescence of UCP-1 (green) and lymphatics (red; LYVE-1) from SQAT depot of Control and AdipoVD following cold exposure and neurotensin receptor inhibition. **(A–E)** *n* = 10 control/*n* = 7 AdipoVD. Blue = DAPI. Bars = 100 µm.

To validate that the reduced thermogenic capacity of AdipoVD mice was specifically due to their elevated neurotensin levels, we tested if inhibiting neurotensin activity could increase cold tolerance. Neurotensin receptor 2 was inhibited for 5 days prior to cold challenge. The body temperature decline of AdipoVD mice over 4 h followed their littermates precisely with neurotensin inhibition (Figure 4A) and no AdipoVD mice needed to be removed from the cold early.

With adipocyte neurotensin signaling inhibition, no significant differences were measured in the expression of browning genes in the BAT tissues of AdipoVD mice and their littermates (Figure 4B). UCP1 immunofluorescence labeling of BAT depots of both Control and AdipoVD tissues demonstrated no marked difference in UCP1 expression while inhibiting neurotensin activity (Figure 4C). Inhibiting neurotensin activity resulted in significantly increased *Cox7a* and *Cox8b* RNA in AdipoVD SQAT compared to controls (Figure 4D). The SQAT depot from AdipoVD mice treated with neurotensin inhibitor looked similar to that of Control tissue in UCP-1 labeling, though little *bona fide* ‘browning’ was present in the short experimental time (Figure 4E). The additional neurotensin produced by LECs in AdipoVD mice was therefore the cause of their body temperature dysfunction demonstrating an anti-thermogenic effect of adipose tissue lymphatic vessels.

## Discussion

The neuropeptide neurotensin has a variety of effects throughout the body but was recently described as a potential anti-thermogenic factor that is highly expressed by lymphatic endothelial cells. Murine adipose tissue, which is highly adaptive to cold exposure, has few lymphatic vessels under normal physiological conditions. In this study we demonstrate that AdipoVD mice, which possess a dense lymphatic vessel network in SQAT and BAT depots, exhibit elevated tissue neurotensin tissue levels. AdipoVD mice were highly susceptible to cold challenge and blocking neurotensin signaling eliminated this effect. We can thus conclude the adipose lymphatic vessels specifically reduce the thermogenic response to cold.

Lymphatic vessels play a crucial role in maintaining tissue homeostasis through transport of macromolecules/lipids, drainage of interstitial fluids, and immune modulation. Lymphangiogenesis, the expansion of lymphatic endothelial density, is often part of the inflammatory response and in most pathologies aids in remediating inflammation (Wiig and Swartz, 2012; Abouelkheir et al., 2017). Adipose tissue inflammation in obesity has been identified as one of the predominant drivers of the metabolic syndrome as the hypoxic environment recruits immune cells perpetuating tissue dysfunction (Rutkowski et al., 2009). In obese adipose tissues levels of the lymphatic growth factors VEGF-C and VEGF-D are elevated, but little lymphangiogenesis occurs in mouse studies (Karaman et al., 2015; Chakraborty et al., 2019). An expanded lymphatic network could, potentially, reduce adipose tissue inflammation in obesity. However, targeting the VEGF-C/D and VEGFR-3 signaling axis appears to have two disparate phenotypes with some studies demonstrating an enhanced metabolic profile while others demonstrate exacerbated adipose inflammation (Chakraborty et al., 2019; Karaman et al., 2015; Karaman et al., 2016). Using AdipoVD mice, here and previously we have demonstrated that increased lymphatic vessels specifically in adipose tissue improved overall glucose metabolism in obesity, increased glycerol flux during lipolysis, and positively altered immune cell populations (Chakraborty et al., 2019). How lymphatic vessels improve tissue function could thus be through their transport roles or through immunomodulation.

Recent studies have identified a new role for lymphatics in the form of tissue paracrine signaling. Cardiac LECs and lymphangiogenesis were identified to secrete and increase reelin and that LEC reelin secretion was key in ameliorating cardiac tissue following injury (Liu et al., 2020). LEC-secreted reelin was also described as important in maintaining intestinal epithelial stem cell niches (Niec et al., 2022). In another stem cell niche, hair follicles, Sosdc1 was identified as another LEC-secreted paracrine factor important in maintaining the microenvironment. Recently, LECs were identified as a potent source of neurotensin production (Li et al., 2021). Neurotensin has been previously studied in a variety of tissue with various effects, typically linked to dopaminergic signaling, and has been demonstrated to produce a hypothermic response (Katz et al., 2004). In a single cell RNA sequencing experiment of human and mouse adipose tissues, Li and others found a small population of LECs (compared to total cells) in adipose tissues but what stood out in these cells was specifically their *Nts/NTS* expression. Using neurotensin inhibition and LEC-specific deletion of Nts, they confirmed that neurotensin was anti-thermogenic and an abundance of this effect was due to LECs; they concluded that sparse lymphatics in adipose tissues permit adaptive thermogenesis (Li et al., 2021). Despite the positive benefits of VEGF-D overexpression and lymphangiogenesis on metabolism in AdipoVD mice, we identify in the current study that increased adipose lymphatics 1) increase tissue neurotensin levels and 2) decrease the thermogenic response. Short-term blockade of the predominate neurotensin receptor in adipocytes, NTSR2, restored temperature control in AdipoVD mice, validating this intriguing paracrine effect. It is not clear from this work whether this effect is mediated by the dense LEC network in AdipoVD brown adipose tissue (BAT being nearly devoid of lymphatics) or activation of browning in WAT. Further depot-specific studies could identify the relative importance of LEC neurotensin to these tissues’ response. Due to rapid temperature decline in AdipoVD mice, the cold exposure time was brief thus limiting many of the classical tissue readouts of adaptive thermogenesis measured by gene or protein expression, such as browning associated genes or WAT UCP1 levels in our study. Longer term cool temperature studies should be performed to identify to what degree this adaptation is impacted in AdipoVD mice as compared to the chronic cool temperature studies conducted previously (Li et al., 2021).

The present work in AdipoVD mice confirms much of the exquisite work by Li and others to demonstrate clear LEC expression of Nts being the predominant driver of this phenomenon. The authors acknowledge that other cell types could still play a role with low levels of Nts elsewhere (blood vessels are *Nts-*positive in their work, for example) or from other Prox1-expressing cells from which *Nts* would be deleted in the Prox1-CreERT2 mouse used (Li et al., 2021). There must be other cellular or systemic effects, however, as beyond the noted direct neurotrophic roles, systemic inhibition of Nts has demonstrated to reduce weight gain, improve metabolism, and lower liver lipid levels so it is possible that in our obese mice, a greater metabolic effect beyond adipose lymphatic-Nts-adipocyte signaling is at play in the corrected thermogenic response with NTSR2 blockade (Li et al., 2016; Wu et al., 2021).

Our findings of significantly elevated neurotensin protein levels present in the adipose tissue once the lymphatic network is greatly expanded reinforces a LEC source for neurotensin, but their contribution to the total amount in the tissue was relatively low. Norepinephrine, key to the sympathetic response to drive thermogenesis, was identified to suppress LEC neurotensin secretion and production (Li et al., 2021). We have previously measured reduced norepinephrine levels in AdipoVD SQAT, thus potentiating an even further limited thermogenic response through both adrenergic signaling and neurotensin’s inhibition of this axis (Chakraborty et al., 2021). We have also previously demonstrated that AdipoVD mouse adipose tissue has an equivalent or even enhanced lipolytic response to a β3-adrenergic agonist, so this is not lacking in the tissue (Chakraborty et al., 2019). AdipoVD mice do, however, feature changes in adipose tissue sympathetic innervation, with changes in neurite branching and density, and demonstrate reduced sympathetic nerve activity upon mechanical stimulation (Chakraborty et al., 2021). The mice utilized in this study were also purposefully high fat diet fed and obese to mimic the ‘healthier’ adipose tissue previously described as opposed to the fibrotic adipose phenotype found in chow-fed mice (Lammoglia et al., 2016). The model and findings cannot, therefore, preclude other neurogenic signaling effects. Whether targeting neurotensin signaling or lymphangiogenesis has translatable potential in the epidemic of obesity thus remains to be seen.

Lymphatic vessels play many roles role in maintaining tissue homeostasis through fluid and macromolecule transport and their critical regulation of the immune response. Recent breakthrough studies have identified novel roles for LECs through paracrine signaling. The discovery of neurotensin as a potent LEC-secreted inhibitor of the thermogenic response in adipose tissue thus opens another exciting chapter into how lymphatic vessels regulate the local tissue environment in health and disease.

## Data Availability

The raw data supporting the conclusions of this article will be made available by the authors, without undue reservation.
