# Supplementary material for: Increased adipose tissue lymphatic vessel density inhibits thermogenesis through elevated neurotensin levels
Source: Front Cell Dev Biol. 2023 Jan 27;11:1100788. doi: 10.3389/fcell.2023.1100788 (PMC9911872; doi:10.3389/fcell.2023.1100788)
Supplement: Supplementary file 1 [file DataSheet1.PDF]

Supplemental Figure 1

A

B

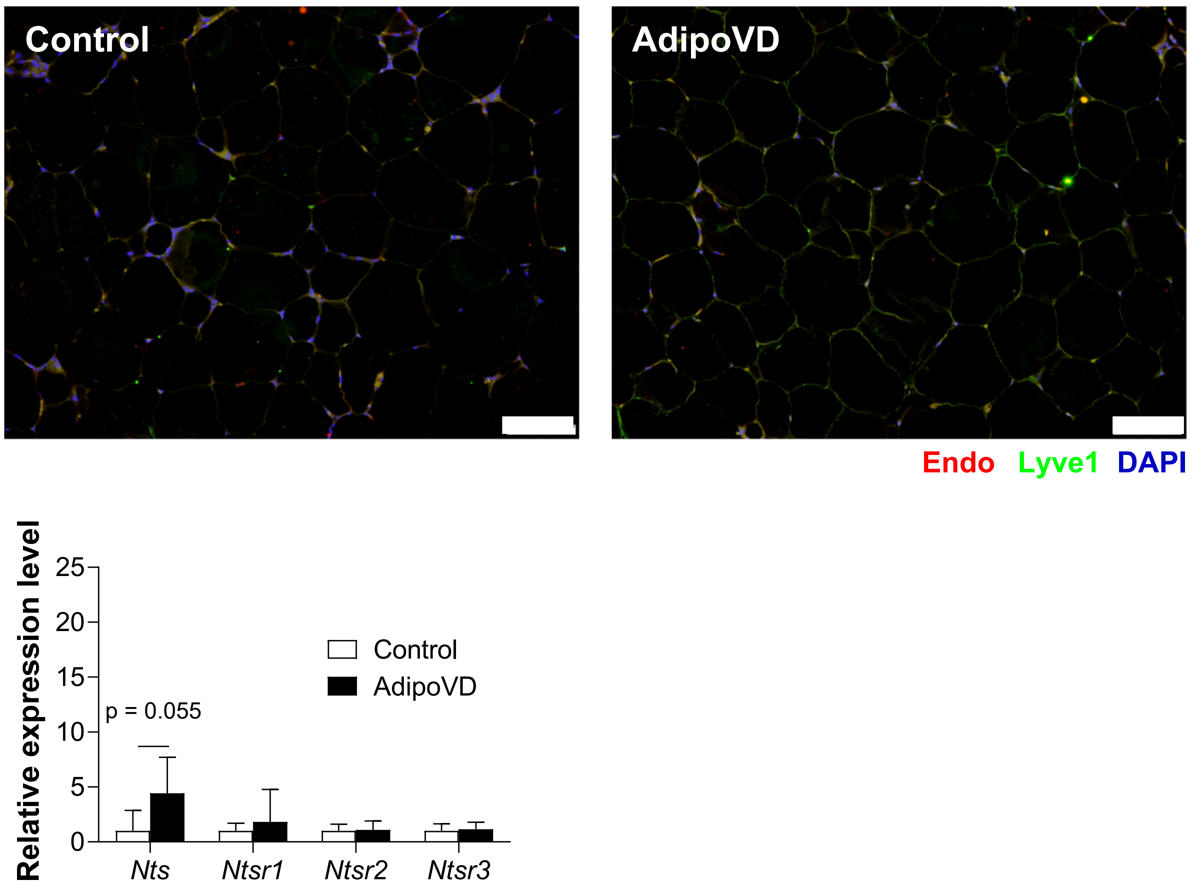

Supplemental Figure 1. Overexpression of VEGF-D expands fails to expand lymphatics significantly in gonadal adipose tissue.

A: Immunofluorescence of lymphatic (green; LYVE-1) and blood (red; Endomucin) in gonadal adipose tissue of Control and AdipoVD mice. **B:** ELISA quantification of neurotensin concentrations from gonadal adipose tissue normalized to tissue protein levels. (**B**) n= 11 for control and AdipoVD. Bars=100 μm.
